# Landscape of Immune Cells Heterogeneity in Liver Transplantation by Single-Cell RNA Sequencing Analysis

**DOI:** 10.3389/fimmu.2022.890019

**Published:** 2022-05-10

**Authors:** Xinqiang Li, Shipeng Li, Bin Wu, Qingguo Xu, Dahong Teng, Tongwang Yang, Yandong Sun, Yang Zhao, Tianxiang Li, Dan Liu, Shuang Yang, Weihua Gong, Jinzhen Cai

**Affiliations:** ^1^Organ Transplantation Center, Affiliated Hospital of Qingdao University, Qingdao, China; ^2^Department of General Surgery, Jiaozuo Women’s and Children’s Hospital, Jiaozuo, China; ^3^The Second Clinical Medical College, Capital Medical University, Beijing, China; ^4^Institute of Organ Donation and Transplantation, Medical College of Qingdao University, Qingdao, China; ^5^Department of Urology Surgery, Peking Union Medical College Hospital, Beijing, China; ^6^Department of Molecular Biology, Medical College, Nankai University, Tianjin, China; ^7^Department of Surgery, Second Affiliated Hospital of School of Medicine, Zhejiang University, Hangzhou, China

**Keywords:** immune, liver tranpslant, single cell RNA sequence, MDSC, CD8 T cell

## Abstract

Rejection is still a critical barrier to the long-term survival of graft after liver transplantation, requiring clinicians to unveil the underlying mechanism of liver transplant rejection. The cellular diversity and the interplay between immune cells in the liver graft microenvironment remain unclear. Herein, we performed single-cell RNA sequencing analysis to delineate the landscape of immune cells heterogeneity in liver transplantation. T cells, NK cells, B cells, and myeloid cell subsets in human liver and blood were enriched to characterize their tissue distribution, gene expression, and functional modules. The proportion of CCR6+CD4+ T cells increased within an allograft, suggesting that there are more memory CD4+ T cells after transplantation, in parallel with exhausted CTLA4+CD8+ T and actively proliferating MKI67+CD8+ T cells increased significantly, where they manifested heterogeneity, distinct function, and homeostatic proliferation. Remarkably, the changes of CD1c+ DC, CADM+ DC, MDSC, and FOLR3+ Kupffer cells increase significantly, but the proportion of CD163+ Kupffer, APOE+ Kupffer, and GZMA+ Kupffer decreased. Furthermore, we identified LDLR as a novel marker of activated MDSC to prevent liver transplant rejection. Intriguingly, a subset of CD4+CD8+FOXP3+ T cells included in CTLA4+CD8+ T cells was first detected in human liver transplantation. Furthermore, intercellular communication and gene regulatory analysis implicated the LDLR+ MDSC and CTLA4+CD8+ T cells interact through TIGIT-NECTIN2 signaling pathway. Taken together, these findings have gained novel mechanistic insights for understanding the immune landscape in liver transplantation, and it outlines the characteristics of immune cells and provides potential therapeutic targets in liver transplant rejection.

## Introduction

Liver transplantation is the only effective way to treat a variety of end-stage liver diseases (1, 2), but rejection is still one of the important reasons leading to the failure of liver transplantation (3). Therefore, studying the mechanism of liver transplant rejection and inducing immune tolerance are the research hotspots in the field of organ transplantation (4). It is well known that cellular immunity plays a leading role in the occurrence and development of rejection (5). It is complex for immune cells to participate in liver transplant rejection or immune tolerance. Different immune cells play different functions in the process of transplant rejection and play different roles in the establishment of immune rejection or immune tolerance (6, 7). A key problem hindering the immune related research of liver transplantation is the lack of understanding of the remodeling of the immune environment in liver transplantation.

Establishing a more comprehensive landscape of immune cells heterogeneity in liver transplantation is crucial to improve understanding of the pathologic physiology. The recent single-cell transcriptome sequencing technologies have brought many new insights into complex physiological mechanisms (8). Single-cell transcriptome sequencing analysis of immune cells allows detailed understanding of these cells in the tissue microenvironment (9). Recently, the new technology has been applied to cancerous and immune cells from patients revealing T cell exhaustion signature and their connection to T cell activation (10, 11). It can help us understand tumor biology regarding collective behavior and regulation of a given tumor cell community (12). In our study, 70,000 cells for the single-cell RNA sequencing analysis were isolated from liver biopsy specimens and PBMC of patients who underwent liver transplantation. We hypothesized that immune cells’ heterogeneity may contribute to a molecular and biological diversity of a cell community in liver transplantation. It should be noted that the interaction between immune cells and hepatocytes in different genetic backgrounds may affect the immune function and metabolic homeostasis of transplanted liver. Fully understanding the process of immune cells remodeling in transplanted liver graft is of great significance to understanding the pathogenesis of postoperative complications and formulate treatment strategies.

To deepen the characterization of the immune cells’ heterogeneity of liver graft after transplantation, we identified some subpopulations of immune cells (CCR6+ CD4+ T, CTLA4+CD8+ T, CD163+ Kupffer, CD4+CD8+FOXP3+ T, LDLR+MDSC), characterized by different spatial distribution in the allograft and gene expression profiles suggesting functional disparity. Therefore, the interaction between donor and recipient to reconstruct the immune microenvironment homeostasis of graft deserves further study. Interestingly, we found that intercellular communication between LDLR+ MDSC and CTLA4+CD8+ T cells interact through TIGIT-NECTIN2 signaling pathway. Collectively, our work provides new light on the cellular compartments that underlie the physiology of the transplanted liver graft and represents a reliable reference for studies on immune microenvironment in human liver transplantation.

## Materials and Methods

### Study Subjects

For baseline patient data (**Supplementary Table S1**), we included patients (n=55) who underwent liver transplantation at Organ Transplantation Center, the Affiliated Hospital of Qingdao University, among November 2020 and October 2021. Data related to patients consisted of gender, age, ABO blood group, and etiology while donor also included organ type, gender, age, ABO blood group, and laboratory data. All liver grafts were voluntarily donated after cardiac death or by living donors and we also included several healthy people as control, which were approved by the Ethics Committee of the Affiliated Hospital of Qingdao University (QYFYWZLL26550). For scRNA-seq, histological analysis, and flow cytometry, all human liver (n=4) and blood samples (n=4) were collected from patients after liver transplantation.

### Human Tissue Cell and PBMC Isolation

After liver puncture tissues were obtained, we placed them into tissue preservation solution immediately. Then, the tissues were transported to be dissociated routinely. Briefly, tissues were cut into approximately 1-2 mm^3^ pieces in the RPMI-1640 medium (Gibco) and digested into single-cell suspensions with the Tumor Dissociation Kit, human (Miltenyi) at 37°C for 60 min. For blood, we collected about 5 ml peripheral blood from one person using anticoagulant tube, which was then centrifuged to prepare PBMC (13). We prepare PBMC using the density gradient centrifugation technique. This is done using an isotonic solution (lymphocyte separator, HISTOPAQUE) with a specific gravity between red blood cells, multinucleated white blood cells, and lymphocytes. The specific gravity of lymphocytes and monocytes is less than or equal to that of the stratified solution, and they float on the surface of the stratified solution after centrifugation. The dissociated cells were subsequently passed through a 70-µm SmartStrainer and centrifuged at 400 g for 5 min. After the supernatant was removed, the pelleted cells were suspended in red blood cell lysis buffer (Miltenyi) to lyse red blood cells. After washing twice with 1× PBS (Gibco), the cell pellets were re-suspended in sorting buffer [PBS supplemented with 2% fetal bovine serum (FBS, Gibco)].

### Library Preparation and Sequencing

Cells were loaded approximately 17,400 cells/chip position using the 10x Chromium Single Cell 3’ Library, Gel Bead & Multiplex Kit and Chip Kit (10x Genomics, V3.1 barcoding chemistry) according to the manufacturer’s instructions. Purified libraries were analyzed by an Illumina nova-seq 6000 sequencer with 150-bp paired-end reads. We applied Cell Ranger (version 3.0.1, 10x Genomics) to generate single-cell data, which processed chromium single-cell RNA-seq outputs to align reads and generate the feature barcode unique molecular identifier (UMI) matrices.

### Single Cell Gene Expression Quantification and Subcluster Delineation

We used the Seurat R package (version 3.2.0) importing raw data to quality control, normalize, scale, and further process data (14). Low quality cells were removed with the following criterion: cells had either fewer than 501 expressed genes, 2001 UMIs, or more than 25% mitochondrial counts. The remaining high-quality cells were normalized and scaled with the default parameters. Highly variable features were identified using FindVariableFeatures function. Then we performed PCA analysis on the scaled data with the determined variable features, dimension reduction, and cluster using FindNeighbors (dims = 1:10) and FindClusters (resolution = 0.5) function. Then we run non-linear dimensional reduction (tSNE) to explore and visualize data.

### Cell Type Determination

We used FindMarkers and FindAllMarkers function to find differentially expressed features of each cluster. Cell types were annotated to known biological types with the canonical marker genes (**Supplementary Table S2**) according to CellMarker database (http://bio-bigdata.hrbmu.edu.cn/CellMarker/) (15) and published articles (16–19). Furthermore, we also used the SingleR package (version 1.2.4) to help identify the cell types (20).

### Functional Enrichment Analysis

After the annotation of each cell type, we employed the functional enrichment analysis to differentially expressed genes between different clusters for Gene Ontology (GO) and KEGG (Kyoto Encyclopedia of Genes and Genomes) analysis to illustrate the biological process and potential function of different cells using clusterProfiler package (version 3.17.0) (21) and org.Hs.eg.db package (version 3.11.4). The p value cutoffs of GO and KEGG were both 0.05. The top 10 terms of results were visualized by barplot or dotplot.

### Pseudotime Analysis

Trajectory analysis was performed using monocle package (version 2.17.0) (22). Including T cells, NK cells, B cells, and myeloid cells, we performed analysis of each group with the following parameters: lowerDetectionLimit=0.5, min_expr=0.1, num_cells_expressed>= 10. For visualization, plot_cell_trajectory function was used to plot the potential trajectory according to pseudotime, seurat clusters, and meta data.

### Cell-Cell Communication Analysis

Cell-to-cell interactions were done using CellChat package (version 1.1.2) (23). The majority of ligand-receptor interactions were mainly on the basis of KEGG signaling pathway database and recent peer-reviewed experimental studies. The main steps of inference of intercellular communications are as follows: (1) Identification of differentially expressed signaling genes. (2) Calculation of ensemble average expression. (3) Calculation of intercellular communication probability.

### Bulk RNA Sequencing Analysis

The transcriptome data for validation were downloaded and processed from the NCBI Gene Expression Omnibus (http://www.ncbi.nlm.nih.gov/geo/) with accession number GSE145780 (7), which measured gene expression by microarrays in 235 liver transplant biopsies, including normal, rejection, and fibrosis samples. We performed further statistic analysis using R software (version 4.0.2).

### Immune Infiltration Estimation

We used CIBERSORT, a method for characterizing complex immune infiltration of tissues according to gene expression profiles, which was developed by Newman et al. (24) from the Alizadeh Lab and was popular in indication of immune infiltration.

### Histological Staining

The fresh liver tissues were fixed with 4% paraformaldehyde and embedded in paraffin, then sectioned in 4 μm in thickness and stained with hematoxylin and eosin (HE), reticular fiber for morphological evaluation, and Masson’s trichrome staining for fibrosis detection.

### Multiplex Immunofluorescence Staining

Briefly, slides were rehydrated with a series of graded ethanol solutions in deionized water. Antigen retrieval was performed, then slides were serially stained with the following antibodies: CD4, CD8, CD11b, CD14, CD15, and FOXP3. Subsequently, Opal IHC Detection Kit (Akoya Biosciences) was applied as a secondary label and antibody signals. Image acquisitions were performed using the Vectra Polaris multispectral imaging platform (Akoya Biosciences), with the entire slide image being scanned and 3-5 representative regions of interest chosen by the pathologist (25).

### Immunohistochemical Staining Analysis

Paraffin sections are routinely dewaxed to hydration, and washed with distilled water. Following incubation in 3% H_2_O_2_ for 10 min, antibodies anti-CD4, CD8, CD11b, CD14, and CD15 were added and incubated at 4°C for overnight. The specimens were incubated with secondary antibodies at 37°Cfor 1 h, followed by diaminobenzidine staining (26).

### Flow Cytometry

Cells were stained using fluorochrome-conjugated antibodies anti-CD3, CD4, CD8a, CD14, CD15, CD45, HLA-DR, and CD11b (BioLegend) according to the protocols provided by the manufacturer. Samples were analyzed using FlowJo software.

### Statistics

For the experimental data, GraphPad Prism 8 (GraphPad Software) was used to perform statistical analyses and graphics production. Data represent mean ± SEM. Results were considered significant when P < 0.05. Differences among the three groups were analyzed using a one-way analysis of variance and Newman-Keuls test for *post-hoc* comparisons.

## Results

### Single Cell Atlas Construction of Human Liver Transplantation

**Figure 1:**
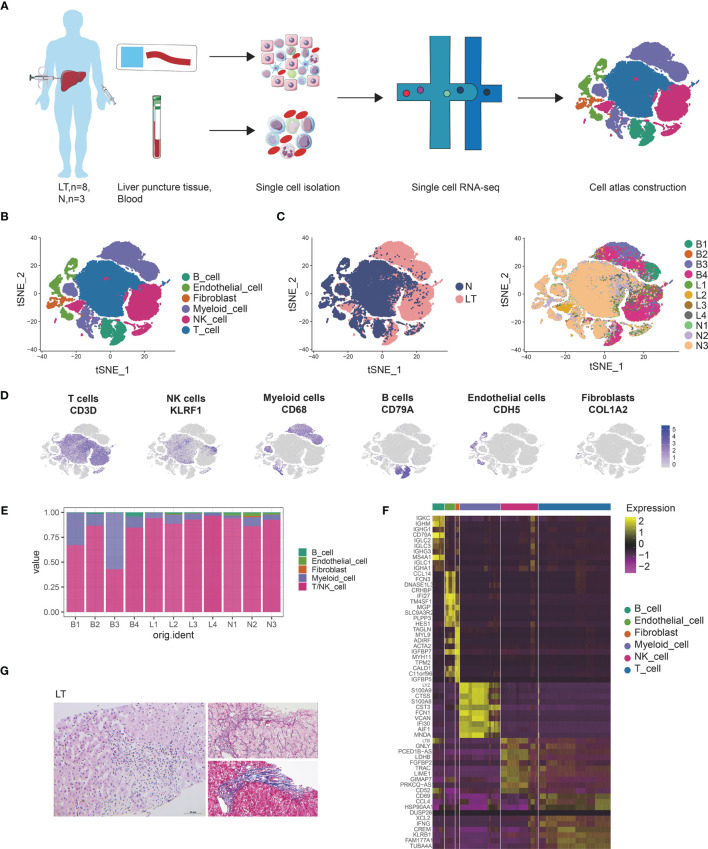
Single cell atlas of human liver transplantation. **(A)** Schematic diagram of scRNA-seq analysis workflow. **(B)** tSNE plots for cell type identification of 68,174 high-quality cells. **(C)** tSNE plot colors by spatial distribution of cells in normal (N) and liver transplantation (LT) tissues. **(D)** Expression of canonical cell markers including CD3D, KLEF1, CD68, CD79A, CDH5, and COL1A2. **(E)** Barplots showing the proportion of cell types in each sample. **(F)** Heatmap showing the top 10 genes of each cell type. **(G)** HE, Masson, and reticular staining of liver transplantation samples.

To reveal the cellular diversity and gene signatures, we performed single cell sequencing from 4 in human liver penetrating tissues and 4 PBMCs from patients after liver transplantation using 10X genomics sequencing, and to discriminate between liver-resident and circulating leucocytes (**Figure 1A**). The clinical meta data and HE staining of samples are shown in **Supplementary Table S1** and **Figure 1G**. We also merged 3 healthy liver data of our previous work. We applied strict filtering on data, including cells expressed genes, unique molecular identifiers (UMIs), and mitochondrial counts (**Supplementary Figures S1A, B**), and further performed normalization and scaling.

We conducted dimensional reduction and clustering including principal component analysis (PCA) and t-distributed stochastic neighbor embedding (t-SNE), of the combined liver tissues and PBMCs with 68,172 high quality cells (**Supplementary Figure S1C**). Compared with PBMCs, cells from liver tissues expressed higher levels of CXCR4 and CCL3L1 genes (**Supplementary Figures S1D–F**), which were liver-resident markers. These clusters were annotated using canonical marker genes (**Supplementary Table S2** and **Figures 1B, D**), across 6 cell lineages, each containing cells from both health and liver transplantation tissues: T cells (27,976 cells, 41.0%, marked with CD3D, CD3E and CD3G), NK cells (14,477 cells, 21.2%, marked with CD7, FGFBP2, and KLRF1), B cells (4534 cells, 6.7%, marked with CD79A, CD79B, and MS4A1), myeloid cells (15,687 cells, 23.0%, marked with CD68, CD14, and CD163), endothelial cells (3944 cells, 5.8%, marked with PECAM1, ICAM2, and ERG), and fibroblasts (1554 cells, 2.3%, marked with COL1A2, COL3A1, and ACTA2). It was found that T, NK, and myeloid cells were dominant in the liver transplantation landscape across samples (**Figures 1C, E**). The differentially expressed genes of each type are shown in **Figure 1F** and **Supplementary Table S3**.

### T, NK Cell Clustering and Subtype Analysis

**Figure 2:**
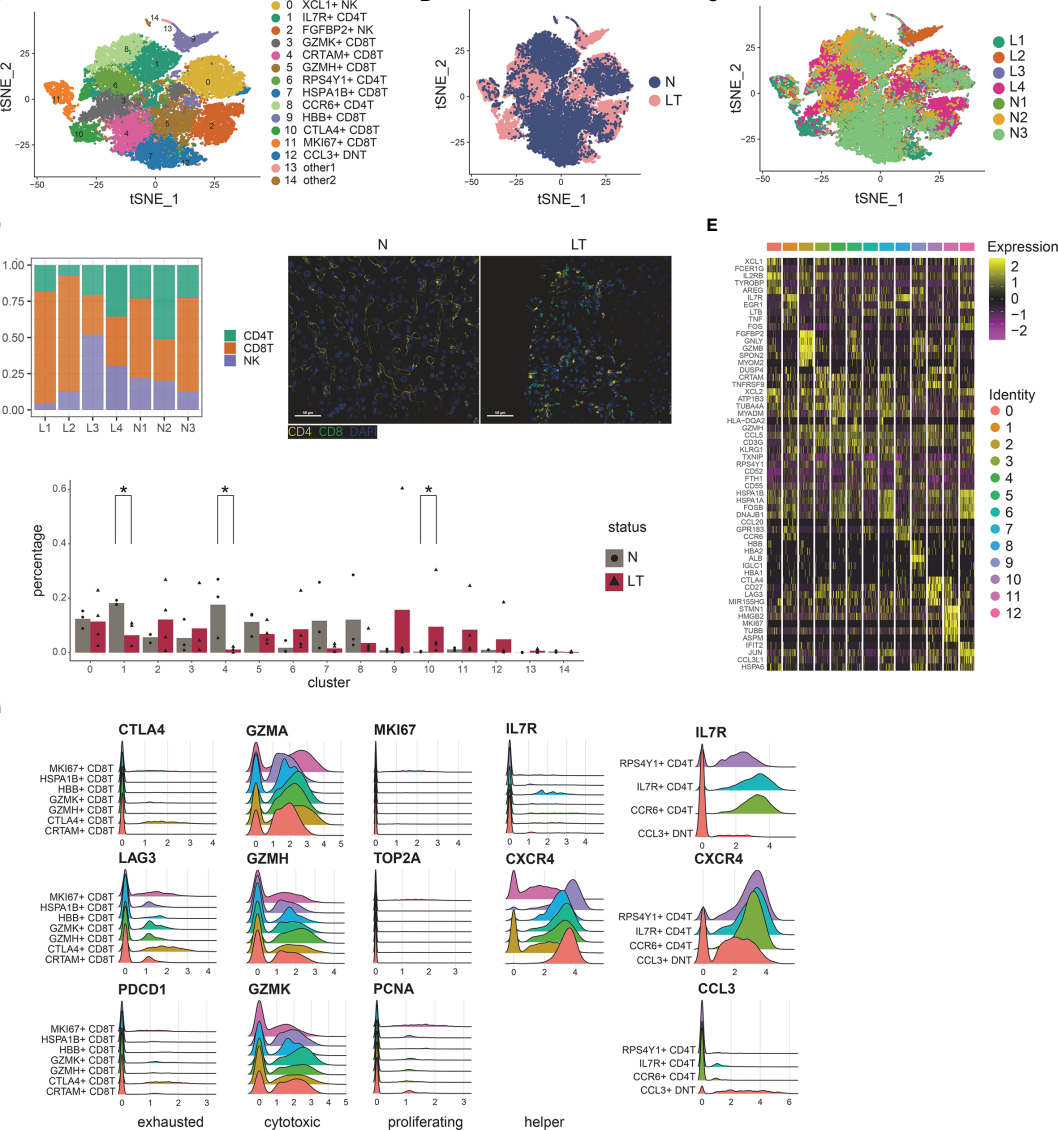
Identifying T and NK cell subpopulations in liver tissue. **(A)** Clustering of 31,300 T and NK cells and cell annotation. **(B)** tSNE plot colors by spatial distribution of cells in normal (n=3) and liver transplantation (n=4) human tissues. **(C)** tSNE plot colors by spatial distribution of cells in 7 samples. **(D)** Barplots showing the proportion of CD4+ T cells, CD8+ T cells, and NK cells in each sample. Immunofluorescence including CD4 and CD8 in normal and liver transplantation. **(E)** Heatmap of each T and NK cells cluster top 5 marker genes. **(F)** Fractions of T, NK cell subpopulations in normal (n=3) and liver transplantation (n=4) samples (* means p < 0.05). **(G)** RidgePlots showing the expression of exhausted, cytotoxic, proliferating, and helper T cells of CD8+ T cells and CD4+ T cells.

To delineate the intrinsic landscape and potential function of the whole T, NK cell of liver tissues, we first re-clustered the 31,300 liver-resident T, NK cells from 7 livers (n=3 health and n=4 liver transplantation), revealing 15 clusters (**Figures 2A–C**). It emerged 7 clusters for CD8+ T cells, 3 clusters for CD4+ T cells, 1 cluster for double negative T cells, and 2 clusters for NK cells (**Figure 2D** and **Supplementary Figures S2A–D**), each cluster containing cells from both healthy and transplanted livers (**Figure 2B**). Cells of the first CD8+ cluster, C3-GZMK-CD8T, specifically expressed marker genes including GZMK, DUSP4, and COTL1 (**Figure 2E** and **Supplementary Table S4**), which was thought to be cytotoxic CD8+ T cells. The second cluster, C4-CRTAM-CD8T, was dominant in healthy tissue significantly (**Figure 2F**), expressing the high levels of CRTAM, HLA-DQA2, and MLF1. The CRTAM gene was involved in regulation of CD8+ T cell regulation, so this cluster was identified as regulatory-like CD8+ T cells. Enrichment analysis also confirmed the phenomenon called “regulation of mRNA metabolic process” (**Supplementary Figure S2E** and **Supplementary Table S4**). However, it did not express traditional Treg markers such as FOXP3 and IL2RA. Moreover, this cluster showed a myeloid phenotype due to the expression of HLA-DQA2 and MLF1. The third cluster, C5-GZMH-CD8T, also showed a cytotoxic phenotype, characterized by specific expression of killing markers including GZMH and GZMA (**Figure 2G**). Furthermore, EGR1 expression pointed to the transcriptional regulator function of this cluster. Particularly, we found 3 CD8+ T clusters with higher proportion in liver transplantation tissues. C9-HBB-CD8T holds plenty of unique genes consisting of HBB, HBA, and ALB, pointing out its specific functions, which needs to be studied further. C10-CTLA4-exhausted CD8T, holding 1495 cells, predominantly composed of cells from liver transplantation tissues, expressed high levels of exhaustion markers CTLA4, PDCD1, and LAG3. C11-MKI67-proliferative CD8T was labelled as the proliferative cluster for the high level of related factors including MKI67 and TOP2A (**Figure 2G**).

CD8+ T clusters showed comparable distribution among patients (**Figures 2A, C, F**). C4- regulatory-CD8T with CRTAM expression and C5-cytotoxic-CD8T with GZMH expression were prevalent in health tissue, while C10-exhausted-CD8T and C11-proliferative-CD8T cells were dominant in liver transplantation tissues. Interestingly, we performed pseudotime analysis to order CD8+ T cells in pseudotime to project the developmental trajectories and found that clusters showed relative time-process, which began with C11-proliferative-CD8T and ended with C10-exhausted-CD8T (**Supplementary Figures S2F, G**). Thus, exhausted CD8+ T cells were highly enriched in the period of the last phase, indicating the T cell state went from proliferation and exhaustion.

Similarly, we identified 3 major CD4+ T clusters (**Figure 2A**), which all expressed high levels of IL7R and LTB (**Supplementary Table S4**). The first cluster, C1-IL7R-CD4T, was significantly dominant in heath tissue, expressing the highest level of IL7R gene, which was identified as T helper cells (**Figure 2F**). Moreover, it especially expressed markers such as TNF and CD40LG, which indicated the activation vs. Type1 immunity. The second cluster, C6-CCR7-CD4T, showed high levels of LEF1, CCR7, and CD62L. CCR7 and CD62L have been shown to mediate the migration of memory T cells, thus this cluster was identified as central memory CD4+ T cells. It also showed the enhancer function for the LEF1. While the last CD4+ cluster, called C8-CCR6-CD4T, similarly expressed CCR7, was identified as another memory T cluster for the special expression of CCR7 and CCR6. With poor expression of CD4 and CD8, we found double negative T cell clusters in the graft tissues, C12-CCL3-DNT, whose marker genes were CCL3L1, IFIT2, and DNAJB4. We identified it as exhausted DNT cluster (**Figures 2A, E**). Two NK clusters (C0-XCL1-NK, C2-FGFBP2-NK) showed different distributions among tissue. The former, C0-XCL1-NK, accounting for the highest proportion of T, NK clusters, showed high levels of XCL1, AREG, and IRF8. While the latter cluster, C2-FGFBP2-NK, expressing FGFBP2, MYOM2, and FCGR3A, was dominant in liver transplantation tissues (**Figures 2A, F** and **Supplementary Table S4**).

### B Cell Clustering and Subtype Analysis

B cells also play great roles in the process of liver transplant rejection. There were 2091 cells further re-clustered and showed functional profile in healthy and liver transplantation tissues. We matched 11 clusters and acquired the differentially expressing genes of each cluster (**Supplementary Figures S2H, I** and **Supplementary Table S5**). C0-RASSF6 expressed high levels of RASSF6, SSPN, and CD82. RASSF6 functions to encode an RAS effector protein which induces apoptosis. Thus, we named this cluster as C0-apoptosis-cluster. C1-ZBTB16, C2-FCRL3, C3-IL6, and C5 might show similar functions for the common high levels of the list of genes, including IGHD, YBX3, IL4R, and FCER2, of which FCER2 is a B cell specific antigen while both IL4R and FCER2 involve the regulation of IgE production. C1-ZBTB16 expressed genes related to cell cycle and cell division, including ZBTB16 and CENPM, simultaneously. C2-FCRL3 holds plenty of unique genes such as FCRL5, FCRL3, and ARL4D, which implicates B cell development. Furthermore, C4-IGHG showed a lot of IGHG family members: IGHG1, IGHG2, IGHG3, and IGHG4. The group of C5-HRK significantly differentially expressed HRK, CCDC191, NEIL1, and PCDH9. Notably, HRK encodes a family of BCL2 proteins, members of which are involved in promoting or inhibiting apoptosis. Thus, C5-HRK was a separate group of apoptosis from the C0-RASSF6. However, the proportion of these two groups of cells in liver transplantation tissue was decreased (**Supplementary Figure S2J**). Both C6-IL32 and C7-IGHA2 expressed T and NK cell related genes, including IL32, NKG7, CD7, GZMK, and GZMA, which are involved in cytotoxicity.

### Distinct Lymphocytes Depict Profile in PBMC

Compared with liver biopsy, peripheral blood is easier to obtain clinically, which has certain potential value in the occurrence and development of transplant rejection and tolerance (27). Thus, we similarly built the liver transplantation atlas of PBMC at the single cell level. By merging 4 healthy data (28), we obtained 46,808 high quality cells after quality control. Dimensionality reduction, cell cluster, and annotation turned out 22 clusters, including 30,465 (65.09%) T/NK cells, 12,557(26.83%) myeloid cells, and 3786 (8.09%) B cells (**Supplementary Figures S3A–C**). We performed further analysis by re-clustering each cell type and detailed annotation (**Supplementary Figure S3D**).

**Figure 3:**
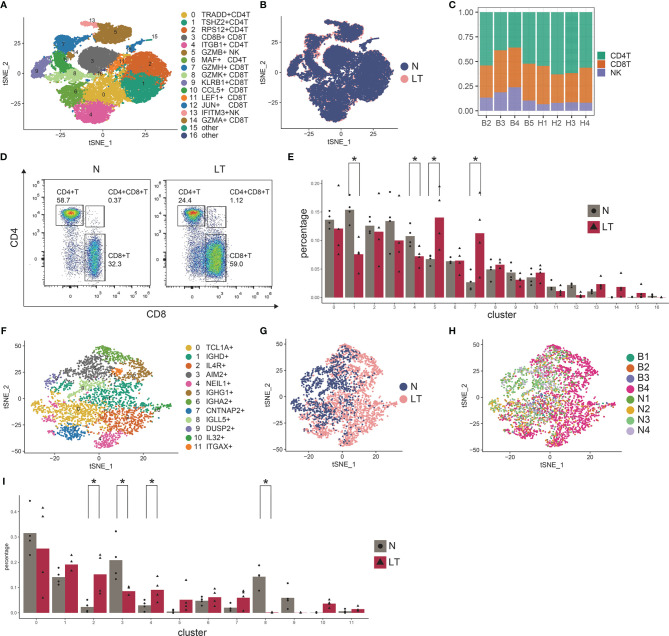
Identifying T and NK cell subpopulations in PBMC. **(A)** Clustering of 30,465 T and NK cells and cell annotation. **(B)** tSNE plot colors by spatial distribution of cells in normal (n=4) and liver transplantation (n=4) human tissues. **(C)** tSNE plot colors by spatial distribution of cells in 8 samples. **(D)** Flow cytometry analysis of CD4+ T cells and CD8+ T cells fraction in normal and liver transplantation tissues. **(E)** Fractions of T, NK cell subpopulations in normal (n=4) and liver transplantation (n=4) samples (* means p < 0.05). **(F)** Clustering of 3786 B cells. **(G)** tSNE plot colors by spatial distribution of cells in normal (n=4) and liver transplantation (n=4) human tissues. **(H)** tSNE plot colors by spatial distribution of cells in 8 samples. **(I)** Fractions of B cell subpopulations in normal (n=4) and liver transplantation (n=4) samples (* means p < 0.05).

Re-clustering T and NK cells obtained 5 CD4+ T cell clusters, 8 CD8+ T cell clusters, and 2 NK clusters (**Figures 3A, B**, **Supplementary Figure S3E**, and **Supplementary Table S6**). We found CD8+ T cells were much more enriched in liver transplantation tissues while CD4+ T cells were relatively lower than those in normal tissue (**Figure 3C**), which was further confirmed by flow cytometry (**Figure 3D**). Similarly, we also identified cytotoxic CD8+ T cells, covering C7-GZMH-CD8T, C8-GZMK-CD8T, C9-KLRB1-CD8T, C10-CCL5-CD8T, and C14-GZMA-CD8T, using canonical markers including GZMA, GZMK, and GZMH (**Supplementary Figure S3F**). Interestingly, we found C7-GZMH-CD8T was significantly dominant in liver transplantation tissue, and it showed NK cell-related genes including NKG7 and FGFBP2. In addition, LAG3 was highly expressed in this cluster, pointing out the potential exhausted state (**Supplementary Figure S3F**). However, we did not find a proliferative CD8+ T cluster. We found C1-TSHZ2-CD4T (TSHZ2 and FHIT highly expressing) and C4-ITGB1-CD4T (ITGB1 and AQP3 highly expressing) in liver transplantation tissue were significantly lower than those in healthy tissue (**Figure 3E**). Importantly, C5-GZMB-NK (GZMB+ SPON2+) is much more prominent in liver transplantation tissue, pointing out that this cluster may play an important role in the process of liver transplant rejection and tolerance.

A total of 3786 B cells were further analyzed (**Figures 3F–H**). We also found several clusters had different proportions between liver transplantation and normal tissue (**Figure 3I**). C2-IL4R-B cell expressed high levels of IL4R, TCL1A, and H1FX, while C4-NEIL1-B cell holds increased expressing level of ALI39020.1, PCDH9, and NEIL1. The proportion of both clusters in liver transplantation tissues was significantly higher than that in healthy tissue. On the contrary, C3-AIM2-B cell and C8-IGLL5-B cell were dominant in healthy tissue. The former expressed S100A11, AIM2, and TLR10 genes while the later expressed IGLL5, IGJ, and HSPA6 genes.

### The Specific Phenotypes of Exhausted CD8 T Cells

**Figure 4:**
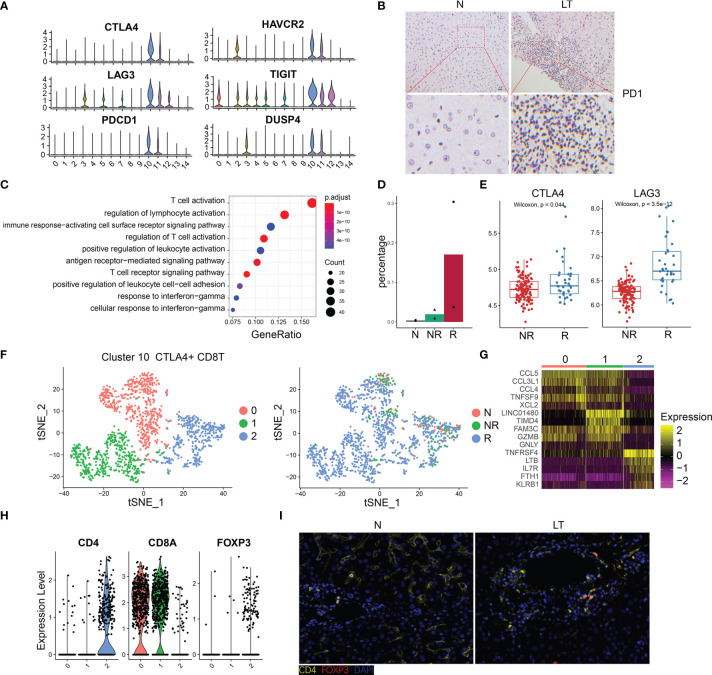
The specific phenotypes of exhausted CD8 T cells. **(A)** Expression of differentially expressed genes in exhausted CD8 T cells. **(B)** Immunohistology of PD1 in normal and liver transplantation tissue. **(C)** Enrichment analysis of Gene Ontology terms for differentially expressed genes in exhausted CD8 T cells. **(D)** Fractions of exhausted CD8 T cells in normal (n=3), stable (n=2), and rejection samples (n=2) after liver transplantation. **(E)** Bulk RNA-seq expression of CTLA4 and LAG3 in stable (N, n=129) and rejection samples (R, n=37) after liver transplantation. **(F)** tSNE plots showing re-clustering of exhausted CD8 T cells. **(G)** Heatmap of each cluster’s top 5 marker genes. **(H)** Expression of CD4, CD8A and FOXP3 among clusters. **(I)** Immunofluorescence including CD4 and FOXP3 in normal and liver transplantation.

The percentage of C10-exhausted-CD8T cells were increased significantly in liver transplantation graft tissues (**Figure 2F**), especially in rejection tissues (**Figure 4D**), which indicated that it may play a significant role in the period of immune tolerance and transplant rejection. Thus, we further explored this cluster, which showed extra markers, such as HAVR2, TIGIT, and DUSP4 (**Figure 4A**). In addition, we validated the high expression of PD-1 using immunohistology of liver transplantation tissues (**Figure 4B**). With identified differentially expressed genes of this cluster, we conducted pathway enrichment analysis. The results showed that these genes were involved in the regulation of T cell activation, T cell receptor signaling pathway, PD-L1 expression, and PD-1 checkpoint pathway (**Figure 4C**, **Supplementary Figure S4A** and **Supplementary Table S4**). The group of C10-exhausted-CD8T cells represented the highest proportion in the rejection group (**Figure 4D**). We confirmed this finding in the bulk RNA-seq, as the expression of exhausted CD8T cells marker genes such as CTLA4 and LAG3 in the rejection group were significantly higher than the non-rejection group (**Figure 4E**, **Supplementary Figure S4B**), confirming the exhausted CD8T cells increased in the rejection group. Moreover, CIBERSORT deconvolution algorithm was used to analyze immune infiltration according to bulk RNA-seq data, which showed that CD8+ T cells were significantly higher in rejection samples than those in stable samples (**Supplementary Figure S4E**).

To further delineate the structure of exhausted CD8+ T cells, we re-clustered this group and acquired 3 detailed clusters (**Figure 4F**). The top 5 marker genes of each cluster are shown in **Figure 4G**. C10-exhausted-CD8T were divided into three groups and cluster 3 expressed higher CD4 and FOXP3 than the others (**Figure 4H**). In addition, we confirmed this finding using multiplex immunofluorescence staining (**Figure 4I**). According to pseudotime analysis, we mentioned above that C10-exhausted-CD8T was highly enriched in the period of the last phase (**Supplementary Figure S2F**). Curious about cluster 2, called CD4+CD8+FOXP3+ T cells, we further speculated on the internal evolution of exhausted CD8 cells. Interestingly, we found CD4+CD8+FOXP3+ T were at the beginning of differentiation (**Supplementary Figures S4C, D**).

### Distinct Myeloid Cells Inhabit the Liver Tissue

**Figure 5:**
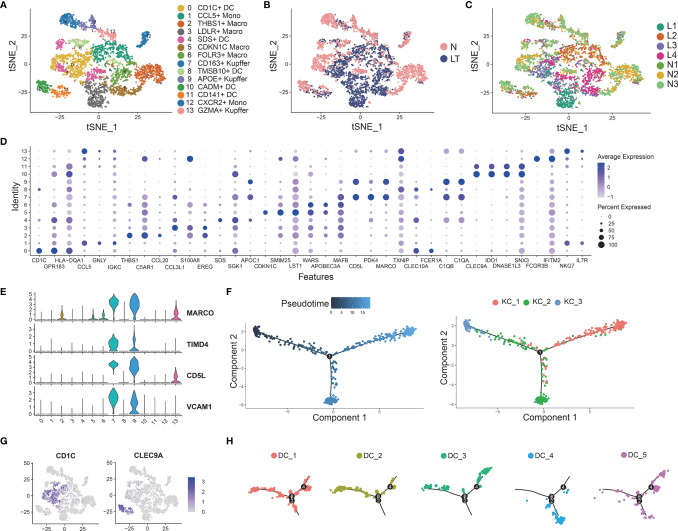
Identifying myeloid cell subpopulations in liver tissue. **(A)** Clustering of 4883 myeloid cells and cell annotation. **(B)** tSNE plots colors by spatial distribution of cells in normal (n=3) and liver transplantation (n=4) human tissues. **(C)** tSNE plots colors by spatial distribution of cells in 7 samples. **(D)** Dotplots of each cluster of myeloid cells’ top 5 marker genes. **(E)** Gene expression of Kupffer cells’ canonical markers across myeloid cells. **(F)** Pseudotime analysis with identified 3 Kupffer groups. **(G)** Gene expression of dendritic cells’ canonical markers across myeloid cells. **(H)** Pseudotime analysis with 5 dendritic cell groups.

We identified 14 clusters by clustering the whole myeloid cells in liver tissue, which were annotated as dendritic cells (DCs), Kupffer cells (KCs), macrophage cells (TMs), and tissue monocytes (Monos) (**Figures 5A–D** and **Supplementary Tables S8****,** **9**). Clusters 7, 9, and 13 were identified as KCs because of the high expression of known markers including MARCO, TIMD4, CD5L, and VCAM1 (**Figures 5A, E**). C7-CD163-Kupffer expressed high levels of classical markers of macrophage such as CD163, which was dominant in health tissue. C9-APOE-Kupffer also holds the larger proportion in healthy tissue with expression of complement C1q family, including C1QA, C1QB, and C1QC. C13-GZMA-Kupffer showed potential cytolytic function, whose markers were GZMA and NKG7, similar to cytotoxic NK cells. Then, we performed pseudotime analysis to 3 KCs, which implied that C13-GZMA-Kupffer was at the end of differentiation (**Figure 5F**). KCs showed similar results to exhausted CD8+ T cells (**Supplementary Figure S2F**). We also found 3 clusters of conventional macrophage cells: C2-THBS1-Macro, C5-CDKN1C-Macro, and C6-FOLR3-Macro. It is worth noting that C2-THBS1-macro was expanded in normal liver significantly.

Clustering of myeloid cells acquired 5 clusters of DCs (**Figures 5A, G**). C0-CD1C-DC showed the largest expression of CD1C, and other markers were FCER1A, CD1E, and SPIB. The second cluster was C4-SDS-DC, with special markers including SDS and A2M. C10-CADM-DC and C11-CD141-DC had similar markers, such as CLEC9A, CADM1, and IDO1, while C10-CADM-DC was enriched in the expression of EGLN3, TACSTD2, and the late state (**Figure 5H** and **Supplementary Figure S5C**).

### The Specific Phenotypes of LDLR+ MDSC

**Figure 6:**
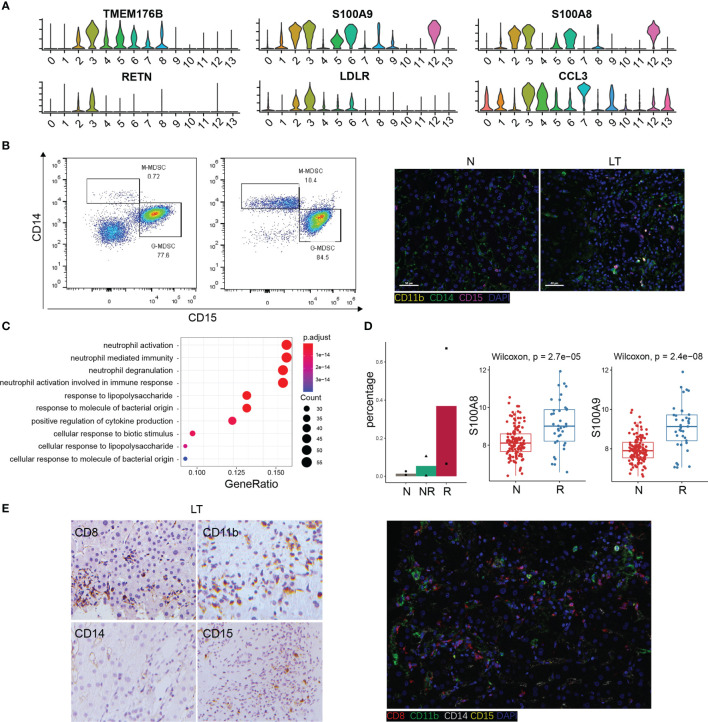
The specific phenotypes of LDLR+ MDSC. **(A)** Expression of differentially expressed genes in LDLR+ MDSC. **(B)** Flow cytometry analysis of MDSC fraction in normal and liver transplantation PBMC, and immunofluorescence including CD11b, CD14, and CD15 in normal and liver transplantation. **(C)** Enrichment analysis of Gene Ontology (GO) terms for differentially expressed genes in LDLR+ MDSC. **(D)** Fractions of LDLR+ MDSC in normal (n=3), stable (n=2), and rejection samples (n=2) after liver transplantation. Bulk RNA-seq expression of S100A8 and S100A9 in stable (n=129) and rejection samples (n=37) after liver transplantation. **(E)** Immunohistology of CD8, CD11b, CD14, and CD15 in liver transplantation tissue, and immunofluorescence including CD8, CD11b, CD14, and CD15 in liver transplantation.

We found C3-LDLR-macro was expanded in liver transplantation tissues (**Supplementary Figure S5A**), so we further explored this cluster. Interestingly, we named it as a group of MDSC with high expression of TMEM176B, S100A8, and S100A9 (**Figure 6A**). The flow cytometry result for PBMC and tissue staining confirmed the result (**Figures 6B, C**). Enrichment analysis indicated that this cluster was involved in neutrophil activation and response to lipopolysaccharide (**Figure 6C**, **Supplementary Figure S6A**), which means plenty of marker genes of this cluster participated in lipid metabolism. In addition, this cluster expressed high levels of RETN and LDLR, which are thought to be strong lipid metabolism related genes. The percentage of LDLR+ MDSC was increased significantly in liver transplantation tissues, especially in rejection tissues (**Figure 6D**). MDSC was recognized as a key factor in inducing immune tolerance and inhibiting transplant rejection. Bulk RNA-seq confirmed MDSC markers, S100A8 and S100A9, were significantly higher in liver transplant rejection tissues than those in stable tissues (**Figure 6D**). We demonstrated these results using immunohistochemistry and fluorescence (**Figure 6E**).

### The Multi-Lineage Interactome in the Liver Tissue

**Figure 7:**
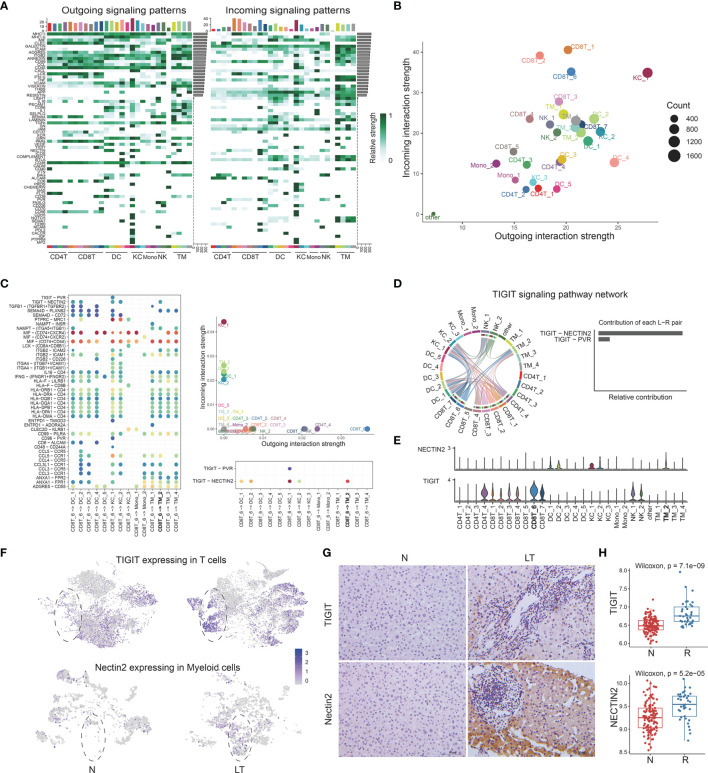
The multi-lineage interactome in the liver tissue. **(A)** Overview of outgoing and incoming signaling patterns among T, NK, and myeloid cell groups in liver transplantation tissue. **(B)** Projecting signaling pathways with bubble plots. Each dot represents the communication network of one signaling pathway. Dot size is proportional to the overall communication probability. **(C)** Dotplots showing the interactions between exhausted CD8+ T cells and myeloid cells in liver transplantation. **(D)** The distribution and contribution of TIGIT signaling pathway network. **(E)** Expression of TIGIT and NECTIN2 among T, NK, and myeloid cells. **(F)** tSNE plots showing the TIGIT expression in exhausted CD8+ T cells and the NECTIN2 expression in LDLR+ macrophage cells. **(G)** Immunohistology of TIGIT and NECTIN2 in normal and liver transplantation tissue. **(H)** Bulk RNA-seq expression of TIGIT and NECTIN2 in stable (n=129) and rejection samples (n=37) after liver transplantation.

After defining the population of T, NK cells, and myeloid cells, we performed cellular communication analysis within liver tissues using CellChat, to infer the intercellular communication network (**Figure 7A**). We constructed the immune populations’ interactions with outgoing and incoming pathways, between ligands, receptors, and cofactors (**Supplementary Table S10**). We visualized the dominant senders and receivers using scatter plot, which unfolded that KC_1, DC_4, and KC_2 showed the top 3 outgoing interaction strengths while CD8T_1, CD8T_2, and CD8T_6 showed the top 3 incoming interaction strengths (**Figure 7B**). Further, to explore how multiple cell types and signaling pathways coordinate, we identified global communication patterns (**Supplementary Figure S7A**). Two patterns of secreting cells basically distinguished T cells and myeloid cells, which were involved in different pathways individually. Three patterns of target cells emerged, noting that DC_2, KC_1, and KC_2 gathered as the same pattern with specific signals.

### TIGIT-NECTIN2 Pathway Matters Between CTLA4+ Exhausted CD8 T Cells and LDLR+MDSC

Having proved that C10-CTLA4-exhausted CD8T was dominant in liver transplantation tissue, with special characteristics, we further explored the interactions between C10-CTLA4-exhausted CD8T and the whole myeloid cells (**Figure 7C**). Importantly, we found the TIGIT-NECTIN2 pathway particularly existed in C10-CTLA4-exhausted CD8T and C3-LDLR-MDSC (**Figure 7C**). C10-CTLA4-exhausted CD8T showed the most powerful outgoing strength in the TIGIT signaling pathway network while LDLR+MDSC placed second to receivers, and TIGIT-NECTIN2 contributed most of TIGIT signal’s ligand-receptor pair (**Figure 7D**). Meanwhile, two clusters showed similar results in the NECTIN signaling pathway network (**Supplementary Figure S7B**). Moreover, we visualized the signaling gene expression distributed among clusters, showing TIGIT and NECTIN2 expressed high levels in C10-CTLA4-exhausted CD8T and C3-LDLR+MDSC separately (**Figure 7E**).

Next, we found the TIGIT gene was highly expressed in T cells from liver transplantation tissue while NECTIN2 genes were dominant in myeloid cells from liver transplantation tissue (**Figure 7F**). In addition, we validated this finding using the immunohistochemical technique (**Figure 7G**). Bulk RNA-seq confirmed TIGIT and NECTIN2 are significantly expressed higher in liver transplant rejection tissues than that in stable tissues (**Figure 7H**). In summary, our exploration of the key ligand-receptor interactions between C10-CTLA4-exhausted CD8T and C3-LDLR+MDSC mentioned the TIGIT-NECTIN2 pathway as an important regulator in the process of transplant rejection and induced immune tolerance.

## Discussion

The differential distribution of immune cells and the heterogeneity of gene expression in various solid organ transplants have been gradually reported (29). In the process of liver transplantation, many immune cells from the recipient enter the donor liver to reshape a new immune microenvironment together with the resident immune cells. However, it is not yet possible to systematically understand the heterogeneity of immune cells, and it is unclear how this difference leads to graft rejection or immune tolerance. Here, we generated a comprehensive single-cell landscape to identify shared cell types, transcriptional programs, and signaling pathways between lymphocyte and myeloid cells in rejection after liver transplantation. It is the first report to demonstrate the presence of biological and transcriptomic heterogeneity of immune cells in human liver transplantation.

First, we analyzed the heterogeneity distribution and gene expression of T, B, and NK cells in liver penetrating tissues and PBMC. According to the immune landscape, we can see that the immune microenvironment is reconstructed and the distribution of immune cells in liver tissues is changed after liver transplantation. Compared with normal liver tissue, the proportion of FGFBP2+ NK, GZMK+CD8+ T, GZMH+CD8+ T, CCR6+CD4+ T, CTLA4+CD8+ T, and MKI67+ CD8 + T cells was increased in transplanted liver. We also found that cytotoxic molecules GZMA, GZMB, GZMH, and NKG7 were highly expressed in these cells. Granzyme A (GZMA) and perforin as markers for rejection in cardiac transplantation (30) and urinary GZMA mRNA levels may entail a diagnostic non-invasive biomarker to distinguish patients with subclinical and acute cellular rejection (31). Biologically, liver penetrating tissues with high rejection level displayed more T cell infiltration and cytotoxicity in comparison with liver penetrating tissues with mild rejection after liver transplantation.

Our data give some novel clues for categorization of both T and NK cell subsets, for T cells, IL7R defines T helper cell, CCR7 defines T naive and Trm cells, CCL3 does for exhausting T cells and CXCR6 does for Trm, CXCR4 defines T helper cells, CXCR3 does for circulating Tem cells, and GZMA/GZMB/GZMK do for cytotoxic T cells. CXCR6, as tissue residence marker, is likely a better marker to define resident memory T cells (Trm), and the proportion of CCR6+CD4+ T cell also increased in the graft, suggesting that there are more memory CD4+ T cells in liver transplantation. Trm maintain immunity in diverse sites, and play an essential role in orchestrating the adaptive immune system through crosstalk (32). Lung transplant recipients exhibiting higher frequencies of persisting donor Trm experienced fewer adverse clinical events compared with recipients with low donor Trm persistence (33).

At the same time, we also found that exhausted CTLA4+CD8+ T cells and actively proliferating MKI67+CD8+ T cells increased significantly in the liver graft. By identifying different cell types in graft immune ecosystem of liver transplantation, we evaluated the relative importance of the different immune checkpoint molecules in human liver transplantation. These exhausted molecules CTLA4, LAG3, HAVCR2, TIGIT, and PDCD1 were highly expressed in the CTLA4+CD8+ T cell. However, our data show that some cell populations are still reduced in the transplanted liver, such as IL-7R+CD4+ T cell, CRTAM+CD8+ T cell, etc.

While analyzing the heterogeneity of lymphocytes and NK cells in transplanted liver and peripheral blood, we also focused on the changes of myeloid cells, such as DC, Kupffer, monocytes, etc. Our data suggest that the number of DC cells in donor liver increases after liver transplantation, and the changes of CD1c+ DC and CADM+ DC cells are more significant. Kupffer cells in the liver showed different trends, FOLR3+ Kupffer cell increased significantly but the proportion of CD163+ Kupffer, APOE+ Kupffer, and GZMA+ Kupffer decreased. CD163+ Kupffer is defined as M2-Kupffer, a macrophage with immunosuppressive function (34), which can inhibit liver transplant rejection (35). The proportion of GZMA+ Kupffer also decreased, highly expressed IL-32, NKG7, GZMK, CCL5, and other genes promoting rejection after organ transplantation. Our analysis reveals a distinct immune ecosystem in liver transplantation, characterized by reduced fractions of classic immunosuppressive cells CD163+ Kupffer and APOE+ Kupffer but the increased fractions of LDLR+ MDSC and CTLA4+ CD8+T cells with highly expressed exhausting molecules and dysfunctional cytotoxicity. These results provide unprecedented insights into the shared programs between normal liver tissue and liver graft immune microenvironment.

The single-cell RNA sequencing analysis had helped us find the MDSC, a subpopulation of myeloid cells (LDLR+, S100A8+, S100A9+, S100A12+, and TMEM176B+) in graft of liver transplantation. For instance, we identified LDLR as a novel marker of activated MDSC, inhibiting liver transplant rejection. MDSC is a group of heterogeneous cells derived from bone marrow, which have the ability to significantly inhibit immune cell response (36). MDSC can induce transplantation immune tolerance by inhibiting antigen nonspecific T cells and promoting Treg proliferation (37). After renal transplantation, M-MDSC in peripheral blood increased immediately, and inhibited the proliferation and effect of CD4+ T and CD8+ T cells, which was conducive to graft survival (38). Through the analysis of intercellular interaction, we found that there was an intercellular communication relationship between LDLR+ MDSC and CTLA4+CD8+ T cell. This data aroused our interest and curiosity, and we conducted a deep analysis of CTLA4+CD8+ T cell. According to the heterogeneity of gene expression, CTLA4+CD8+ T cell was divided into three subgroups, and we were surprised to find that there were a group of CD4+CD8+FOXP3+ T cells. To the best of our knowledge, this is the first report of heterogeneity in the LDLR+ MDSC and CTLA4+CD8+ T cell in human liver transplantation.

Through pseudotime analysis, we believe that, in the process of gradual infiltration, CD8+ T cell is regulated by several factors, then CD8+ T cells increase CD4 gene expression and the phenotype of regulatory T gradually increase the expression of transcription factor FOXP3 to differentiate into CD4+CD8+FOXP3+ T cells. Interestingly, CD4+CD8+ T cells have recently been associated with multiple diseases, and cells can be suppressive or cytotoxic, depending on conditions (cancer, HIV, systemic sclerosis) (39–41). Although some studies support mature CD4+ T cells as the source of CD4+CD8+ T cells (42), evidence of CD8 as the origin lineage of CD4+CD8+ T cell also exists (43). The expression of CD4 on CD8+ T cells is considered to be mainly related to the activation of naive rather than memory CD8+ T cells (44). However, there are few reports on the phenotype and function of CD4+CD8+FOXP3+ T cells, especially in the field of organ transplantation.

An unexpected finding was that CD4+CD8+FOXP3+ T cell was present in the liver tissues of the rejection group persisted throughout the periods of high levels of rejection. How specific CD4+CD8+FOXP3+ T cell populations contribute to liver transplantation is a topic of ongoing discussion. Our findings may constitute a first step toward the identification of new therapeutic targets in rejection after liver transplantation. Interestingly, we analyzed the intercellular communication between LDLR+ MDSC and CTLA4+CD8+ T cells interact through the TIGIT-NECTIN2 signaling pathway. We also observed the higher expression of TIGIT and NECTIN2 signaling components enriched with MDSC, Kupffer cells, and T cells of liver graft, suggesting the importance of TIGIT-NECTIN2 signaling in maintenance of transplantation immunity ecosystem. TIGIT on T cells belongs to immunoglobulin superfamily receptors, which interacts with NECTIN molecules. The signals formed by TIGIT-NECTIN2 contribute to regulation of immune functions. Blocking TIGIT/NECTINs may have a potential effect on immunotherapy (45, 46). Similar to previous reports, tumor-associated macrophages suppress tumor T cell infiltration and TIGIT-NECTIN2 interaction regulates the immunosuppressive environment in HBV-associated HCC (47). The validation of these pathways greatly contributes to better resolution of CD163+ Kupffer, LDLR+ MDSC, and CD4+CD8+FOXP3+ T cell. Taken together, among the subpopulations of LDLR+ MDSC was specifically engaged in immune-suppressive interactions with CTLA4+CD8+ T cell.

In summary, we try to delineate the underlying high-resolution multifaceted landscapes, and provide new light on the cellular compartments that underlie the physiology of the liver transplantation and represents a reliable reference for studies on immune microenvironment in human liver transplantation. Given the wide range of functional contributions of immune cells for liver transplant rejection, a better understanding of the heterogeneity and subpopulations of LDLR+MDSC and CTLA4+CD8+ T, especially CD4+CD8+FOXP3 T cells may help to identify novel therapeutic targets to treat rejection after liver transplantation.

## Data Availability Statement

The datasets presented in this study can be found in online repositories. The name of the repository and accession number can be found below: NGDC Genome Sequence Archive (https://ngdc.cncb.ac.cn/gsa-human/); HRA002091.

## Ethics Statement

The studies involving human participants were reviewed and approved by the ethics committee of Affiliated Hospital of Qingdao University. Written informed consent to participate in this study was provided by the participants’ legal guardian/next of kin.

## Author Contributions

JC, SL, and WG contributed to the research design. XL, BW, DT, and QX collected the clinical data. DT and XL performed multiplex immunofluorescent assays and immunocytochemistry. TY, YS, XL, TL, DL, and BW contributed to the molecular biology experiment and pathology experiment operation. SL, SY, YZ, and XL contributed to the data management and statistical analyses. JC, SL, and XL wrote the manuscript. All authors contributed to the article and approved the submitted version.

## Funding

This work was supported by the National Natural Science Foundation of China (No. 81670600) and clinical medicine + X of the Affiliated Hospital of Qingdao University.

## Conflict of Interest

The authors declare that the research was conducted in the absence of any commercial or financial relationships that could be construed as a potential conflict of interest.

## Publisher’s Note

All claims expressed in this article are solely those of the authors and do not necessarily represent those of their affiliated organizations, or those of the publisher, the editors and the reviewers. Any product that may be evaluated in this article, or claim that may be made by its manufacturer, is not guaranteed or endorsed by the publisher.

## Supplementary Material

Supplementary Figure 1 Quality control, cluster, and liver-resident gene expression. **(A)** NFeature_RNA, nCount_RNA, and percent.mt (refers to the number of genes, UMIs, and the percentage of mitochondrial genes, respectively) used for quality control. **(B)** Visualization of nCount_RNA-percent.mt relationship and nCount_RNA-nFeature_RNA relationship using FeatureScatter. **(C)** Clustering of 69,816 high-quality cells. **(D)** CXCR4 and CCL3L1 expression between blood and liver across cell types.

Supplementary Figure 2 T, NK, and B cells annotation and analysis. **(A)** tSNE plots showing the expression of the CD4 gene (white to blue). **(B)** tSNE plots showing the expression of the CD8 gene. **(C)** tSNE plots showing the expression of the KLRF1 gene. **(D)** Clustering of 31,300 T and NK cells and annotation basically. **(E)** Pathway enrichment analysis of differentially expressed genes of C4-CRTAM-CD8T. **(F)** Pseudotime analysis of CD8+ T cells in liver tissue. **(G)** Pseudotime analysis showing CD8T_6 and CD8T_7 in liver tissue. **(H)** Clustering of 2091 B cells among normal and liver transplantation samples. **(I)** Top 5 marker genes’ expression of each B cluster. **(J)** Fractions of B cell subpopulations in normal (n=3) and liver transplantation (n=4) samples.

Supplementary Figure 3 Clustering and annotation of the whole cells in PBMC. **(A)** tSNE plots showing the clustering of 46,808 high quality cells in PBMC. **(B)** tSNE plots showing the cells’ distribution among normal and liver transplantation samples. **(C)** tSNE plots showing the annotation of cells. **(D)** tSNE plots showing the gene expression of CD4, CD8, and KLRF1 (white to blue). **(E)** tSNE plot colors by spatial distribution of T and NK cells in 8 samples. Clustering of 30,465 T and NK cells and annotation basically. **(F)** Violin plots showing the expression of selected cytotoxic, exhausted, proliferative marker genes.

Supplementary Figure 4 Clustering and analysis of myeloid cells in liver tissue. **(A)** Fractions of myeloid cell subpopulations in normal (n=3) and liver transplantation (n=4) samples. **(B)** Clustering of 4883 myeloid cells and annotation basically. **(C)** Pseudotime analysis with identified 5 dendritic cell groups (colored by pseudotime).

Supplementary Figure 5 Further analysis of exhausted CD8+ T cells. **(A)** Enrichment analysis of Kyoto Encyclopedia of Genes and Genomes (KEGG) terms for differentially expressed genes in exhausted CD8 T cells. **(B)** Bulk RNA-seq expression of PDCD1 in stable (N, n=129) and rejection samples (R, n=37) after liver transplantation. **(C)** Pseudotime analysis with identified 3 subgroups of exhausted CD8+ T cells (colored by pseudotime). **(D)** Pseudotime analysis with identified 3 subgroups of exhausted CD8+ T cells (colored by clusters). **(E)** Estimated proportion of CD8+ T cells among stable (N, n=129) and rejection (R, n=37) samples after liver transplantation using CIBERSORT algorithm.

Supplementary Figure 6 The multi-lineage communication patterns and inferred signals between LDLR+ macro and T cells. **(A)** Outgoing communication patterns of secreting cells (left) and incoming communication patterns of target cells (right). **(B)** Dotplots showing the interactions between LDLR+ macro cells and T cells in liver transplantation (left). The distribution and contribution of NECTIN signaling pathway network (right).

Supplementary Figure 7 Enrichment analysis of KEGG terms for differentially expressed genes in LDLR+ MDSC. **(A)** Enrichment analysis of GO terms for differentially expressed genes in LDLR+ MDSC.
