# Supplementary figures and images for: Landscape of Immune Cells Heterogeneity in Liver Transplantation by Single-Cell RNA Sequencing Analysis
Source: Front Immunol. 2022 May 10;13:890019. doi: 10.3389/fimmu.2022.890019 (PMC9127089; doi:10.3389/fimmu.2022.890019)

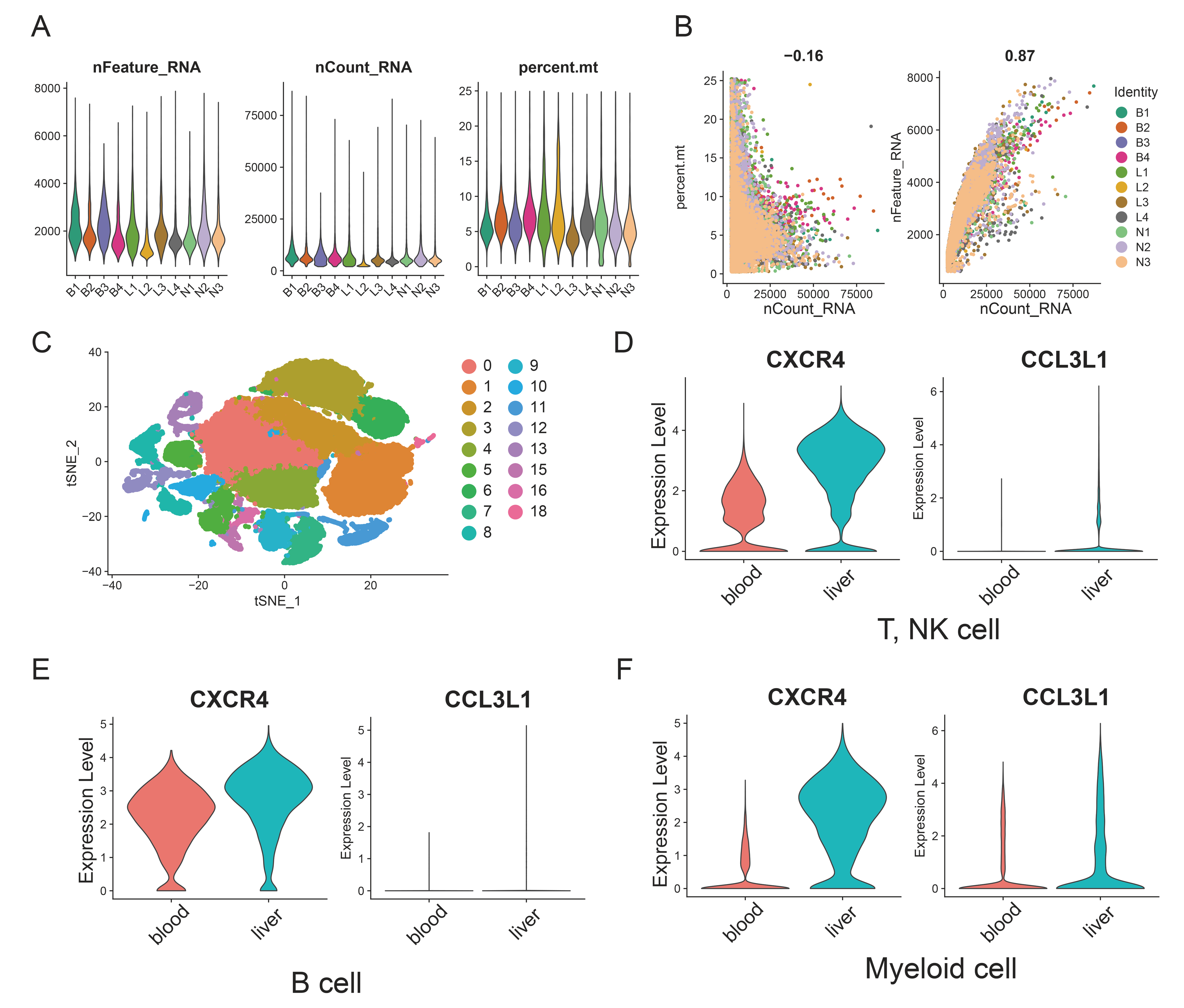

Supplement: Supplementary Figure 1 — Quality control, cluster, and liver-resident gene expression. (A) NFeature_RNA, nCount_RNA, and percent.mt (refers to the number of genes, UMIs, and the percentage of mitochondrial genes, respectively) used for quality control. (B) Visualization of nCount_RNA-percent.mt relationship and nCount_RNA-nFeature_RNA relationship using FeatureScatter. (C) Clustering of 69,816 high-quality cells. (D) CXCR4 and CCL3L1 expression between blood and liver across cell types. [file Image_1.tiff]

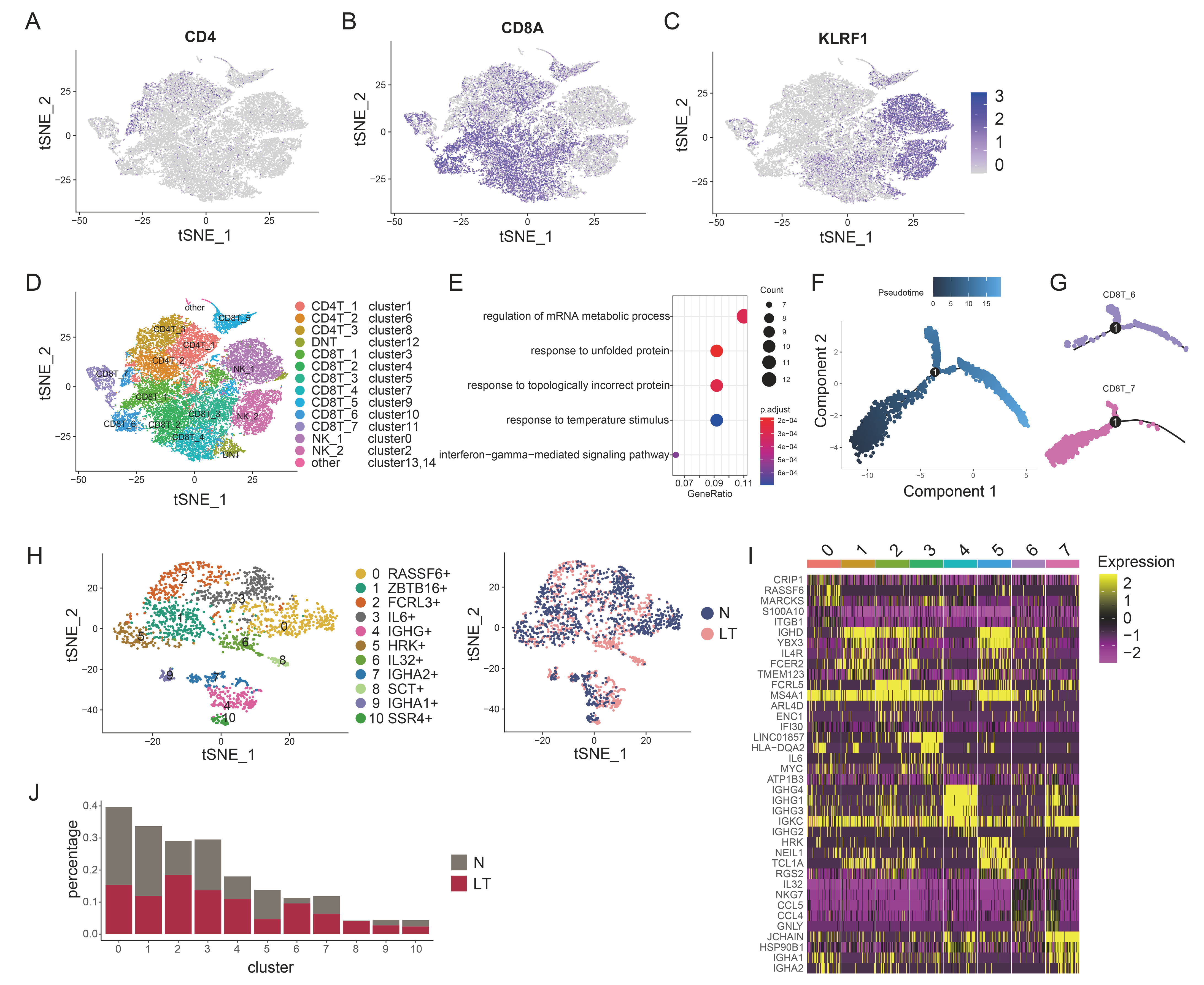

Supplement: Supplementary Figure 2 — T, NK, and B cells annotation and analysis. (A) tSNE plots showing the expression of the CD4 gene (white to blue). (B) tSNE plots showing the expression of the CD8 gene. (C) tSNE plots showing the expression of the KLRF1 gene. (D) Clustering of 31,300 T and NK cells and annotation basically. (E) Pathway enrichment analysis of differentially expressed genes of C4-CRTAM-CD8T. (F) Pseudotime analysis of CD8+ T cells in liver tissue. (G) Pseudotime analysis showing CD8T_6 and CD8T_7 in liver tissue. (H) Clustering of 2091 B cells among normal and liver transplantation samples. (I) Top 5 marker genes’ expression of each B cluster. (J) Fractions of B cell subpopulations in normal (n=3) and liver transplantation (n=4) samples. [file Image_2.tiff]

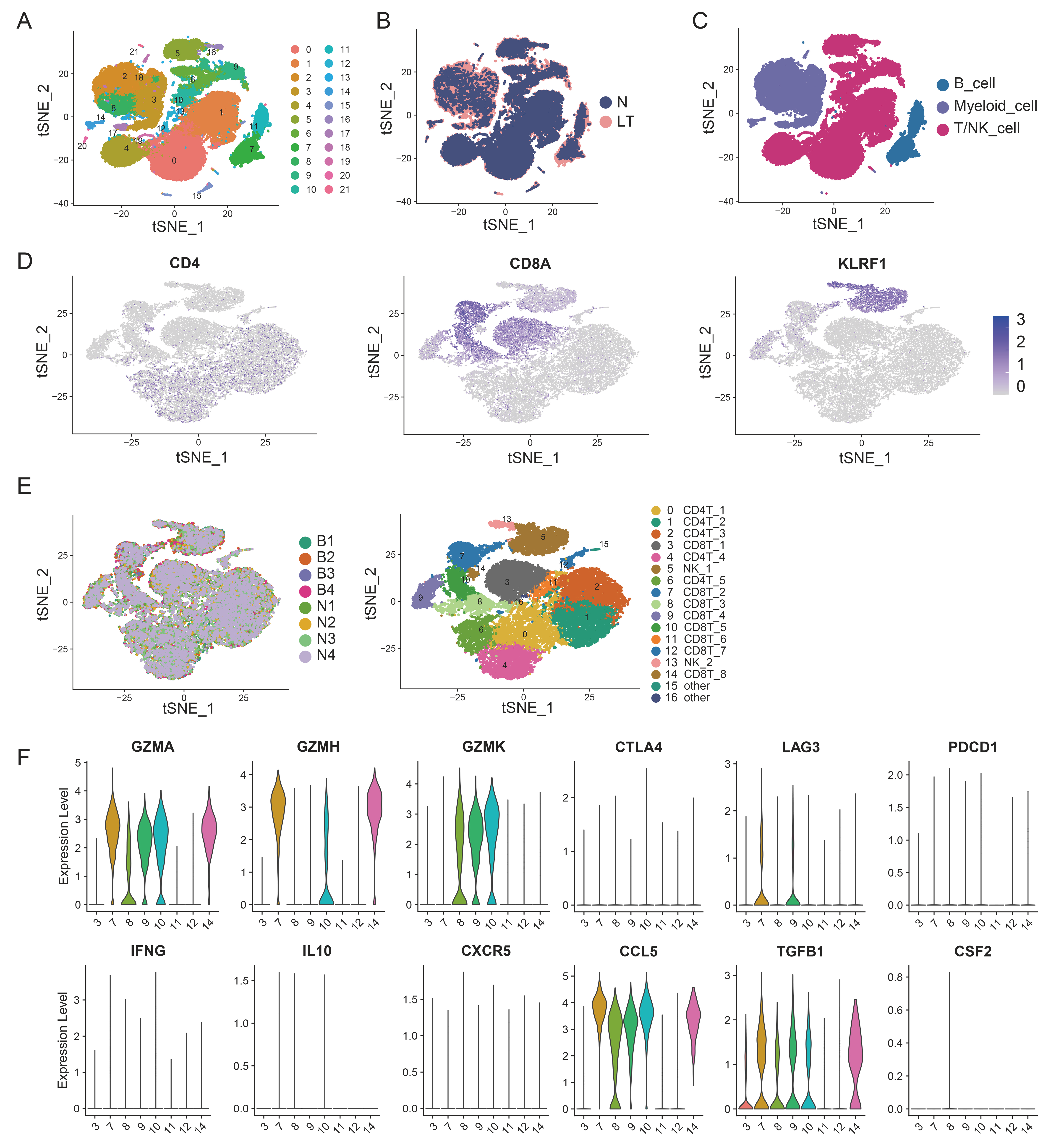

Supplement: Supplementary Figure 3 — Clustering and annotation of the whole cells in PBMC. (A) tSNE plots showing the clustering of 46,808 high quality cells in PBMC. (B) tSNE plots showing the cells’ distribution among normal and liver transplantation samples. (C) tSNE plots showing the annotation of cells. (D) tSNE plots showing the gene expression of CD4, CD8, and KLRF1 (white to blue). (E) tSNE plot colors by spatial distribution of T and NK cells in 8 samples. Clustering of 30,465 T and NK cells and annotation basically. (F) Violin plots showing the expression of selected cytotoxic, exhausted, proliferative marker genes. [file Image_3.tiff]

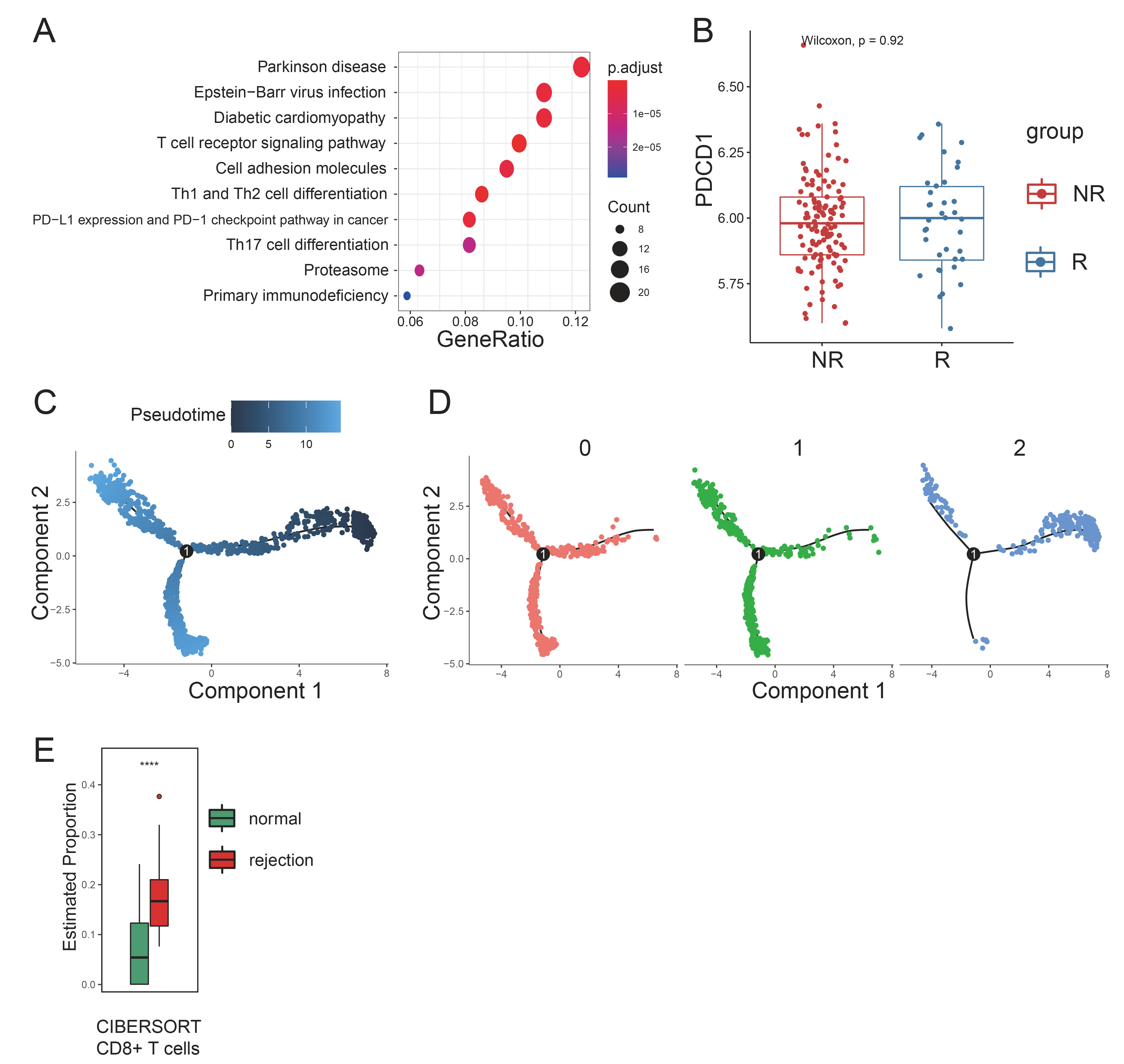

Supplement: Supplementary Figure 4 — Clustering and analysis of myeloid cells in liver tissue. (A) Fractions of myeloid cell subpopulations in normal (n=3) and liver transplantation (n=4) samples. (B) Clustering of 4883 myeloid cells and annotation basically. (C) Pseudotime analysis with identified 5 dendritic cell groups (colored by pseudotime). [file Image_4.tiff]

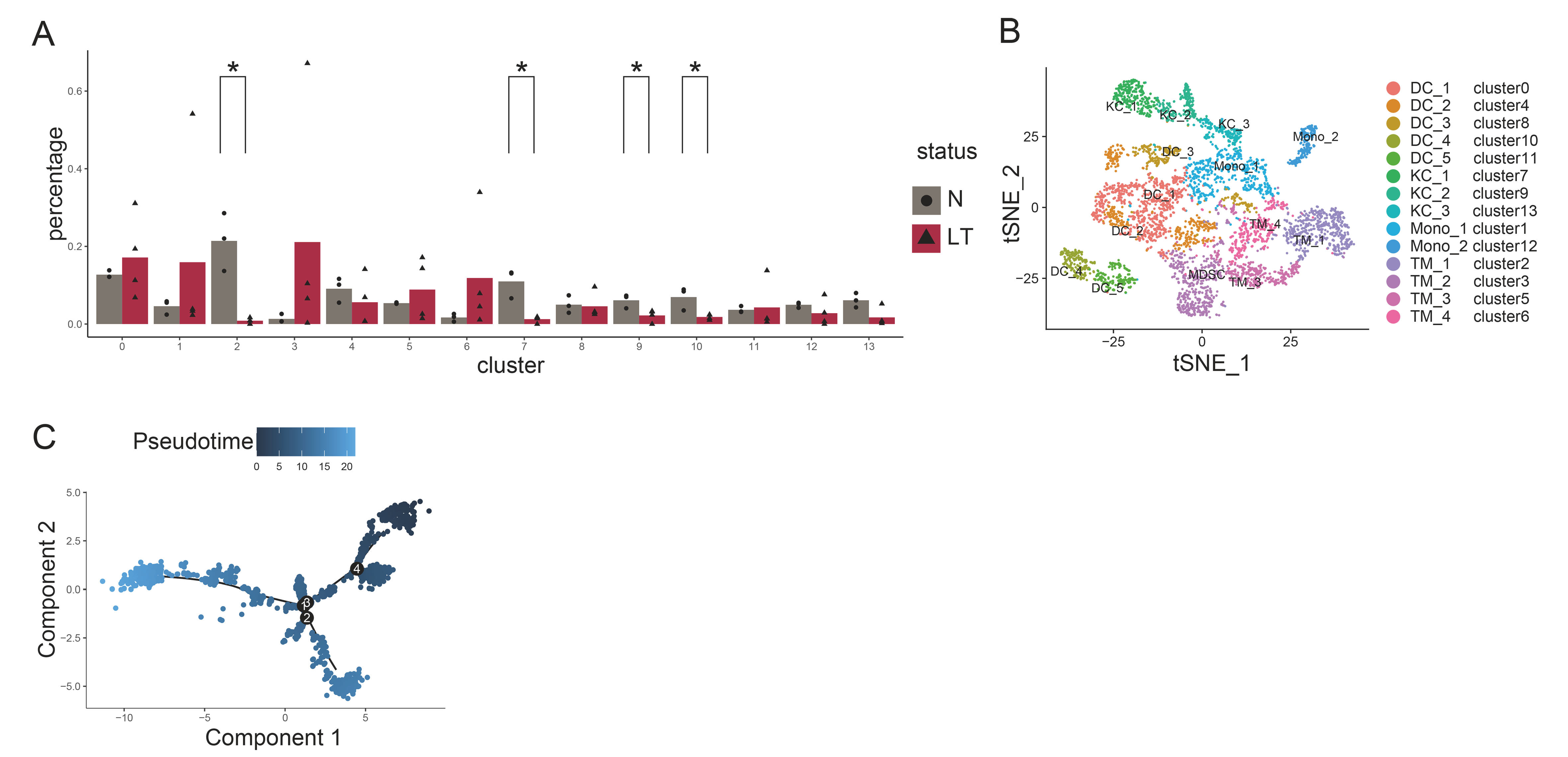

Supplement: Supplementary Figure 5 — Further analysis of exhausted CD8+ T cells. (A) Enrichment analysis of Kyoto Encyclopedia of Genes and Genomes (KEGG) terms for differentially expressed genes in exhausted CD8 T cells. (B) Bulk RNA-seq expression of PDCD1 in stable (N, n=129) and rejection samples (R, n=37) after liver transplantation. (C) Pseudotime analysis with identified 3 subgroups of exhausted CD8+ T cells (colored by pseudotime). (D) Pseudotime analysis with identified 3 subgroups of exhausted CD8+ T cells (colored by clusters). (E) Estimated proportion of CD8+ T cells among stable (N, n=129) and rejection (R, n=37) samples after liver transplantation using CIBERSORT algorithm. [file Image_5.tiff]

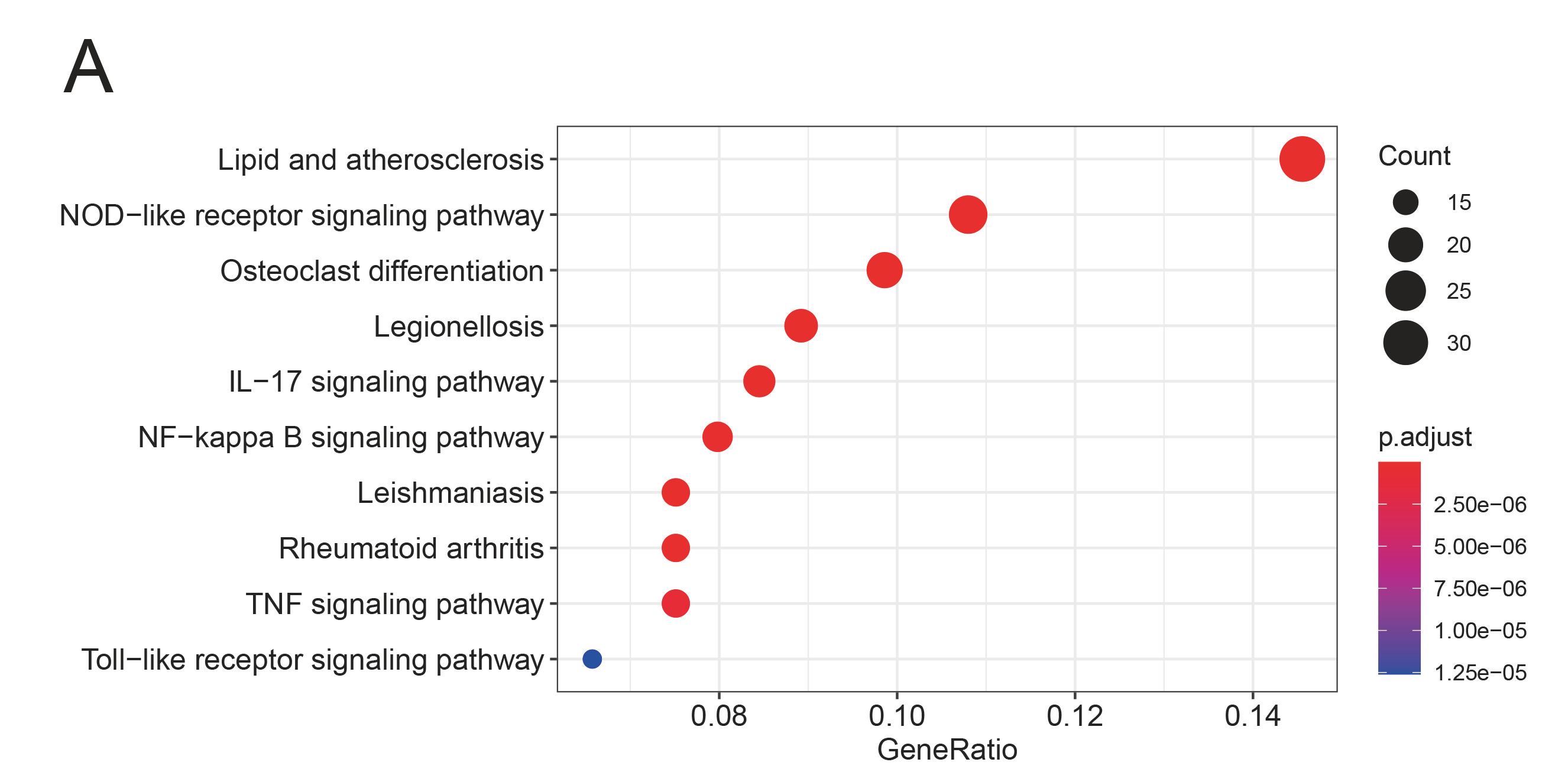

Supplement: Supplementary Figure 6 — The multi-lineage communication patterns and inferred signals between LDLR+ macro and T cells. (A) Outgoing communication patterns of secreting cells (left) and incoming communication patterns of target cells (right). (B) Dotplots showing the interactions between LDLR+ macro cells and T cells in liver transplantation (left). The distribution and contribution of NECTIN signaling pathway network (right). [file Image_6.tiff]

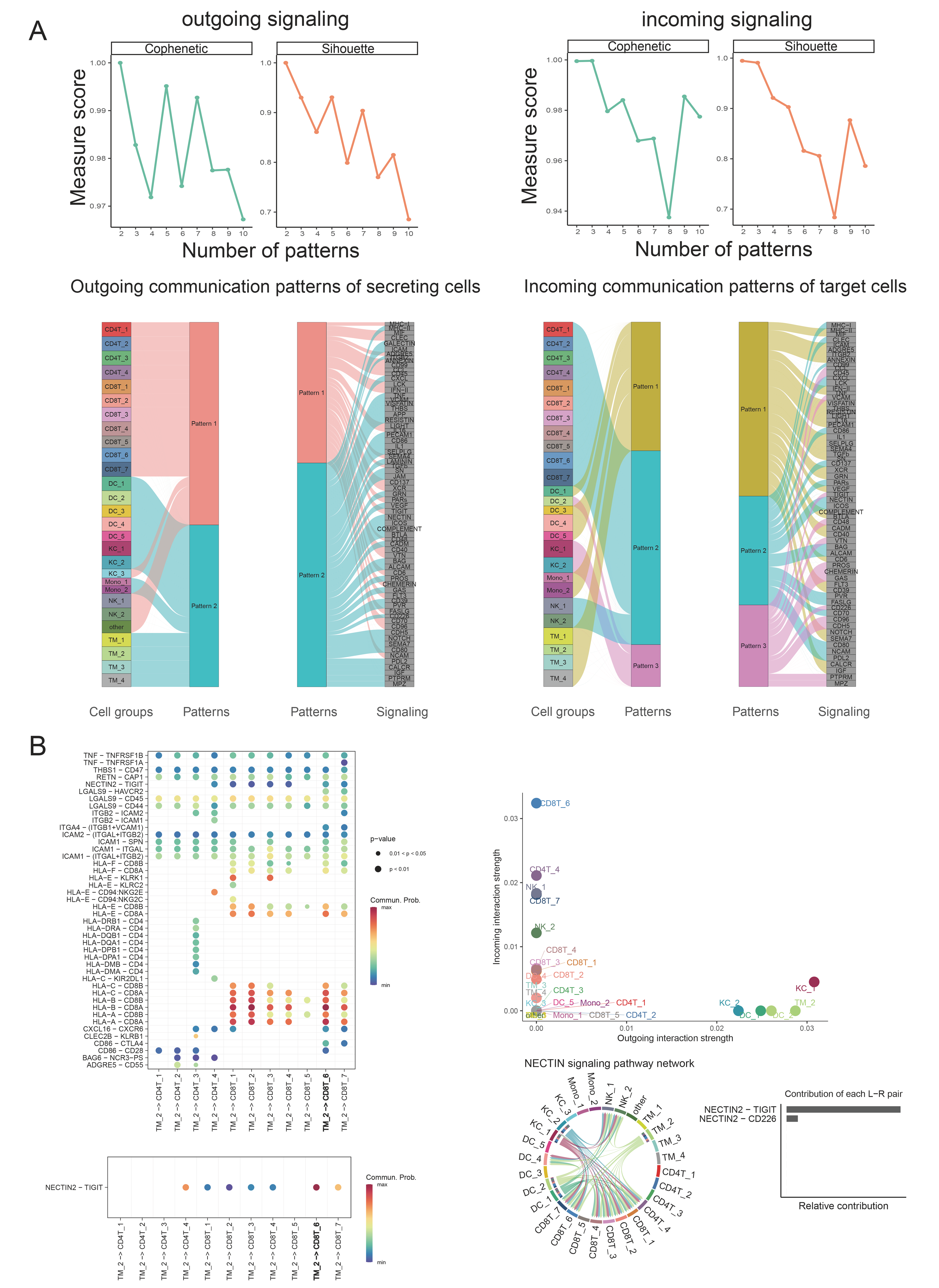

Supplement: Supplementary Figure 7 — Enrichment analysis of KEGG terms for differentially expressed genes in LDLR+ MDSC. (A) Enrichment analysis of GO terms for differentially expressed genes in LDLR+ MDSC. [file Image_7.tiff]
